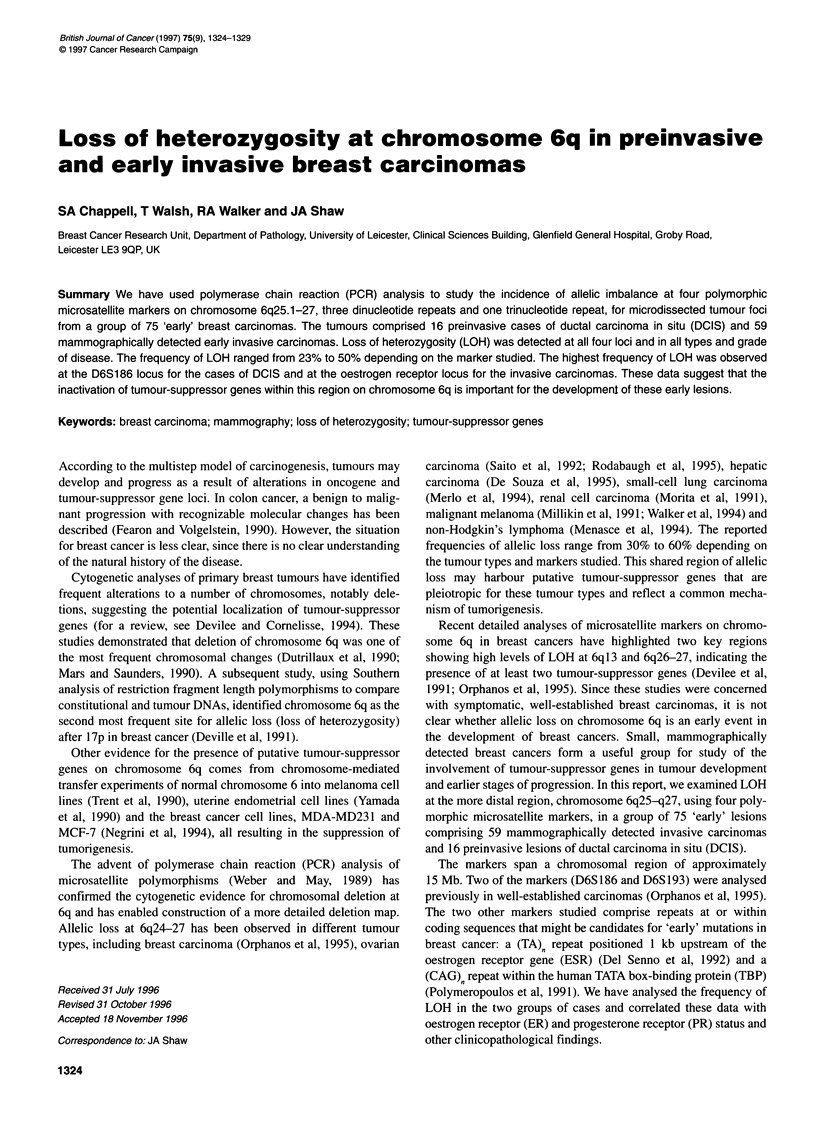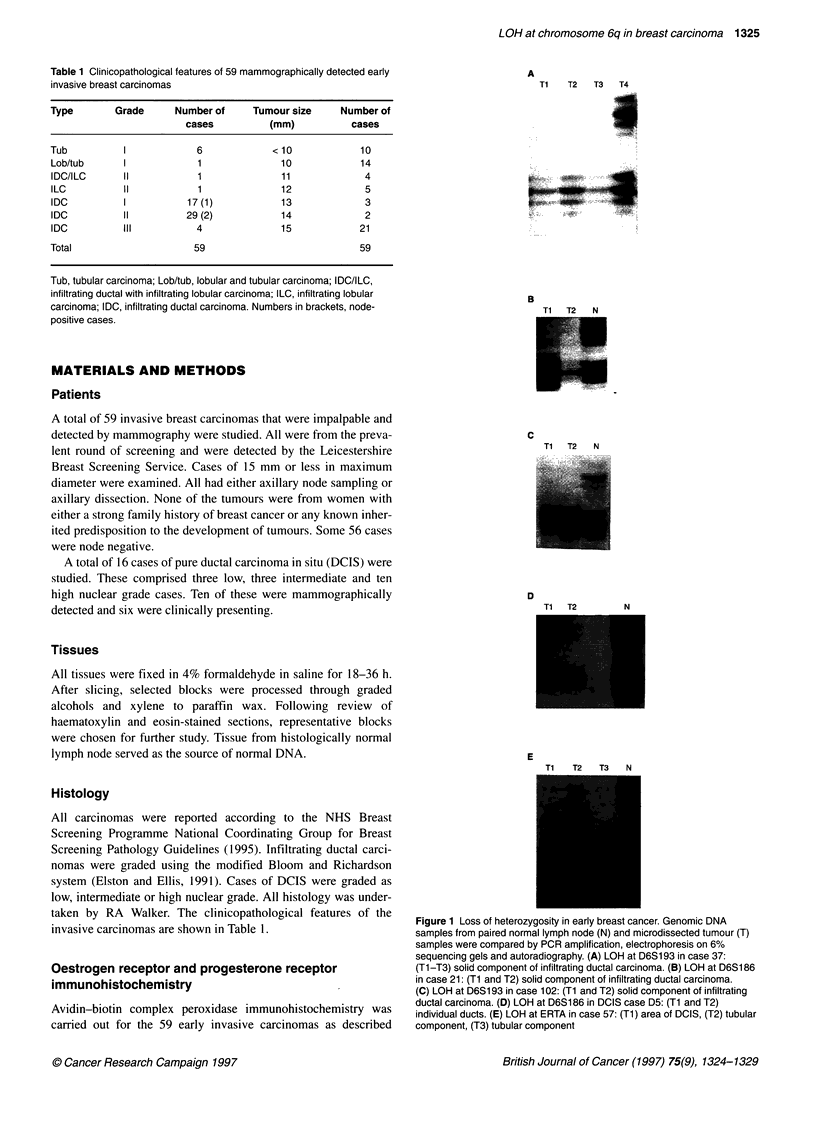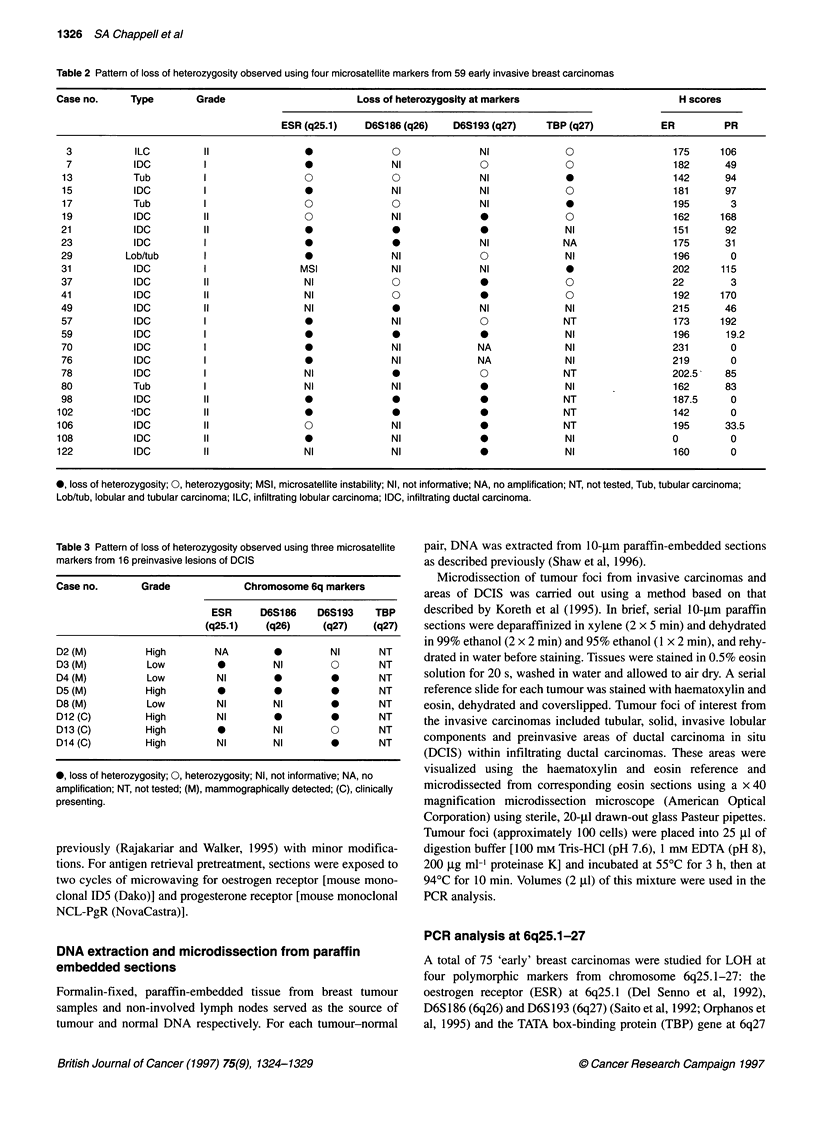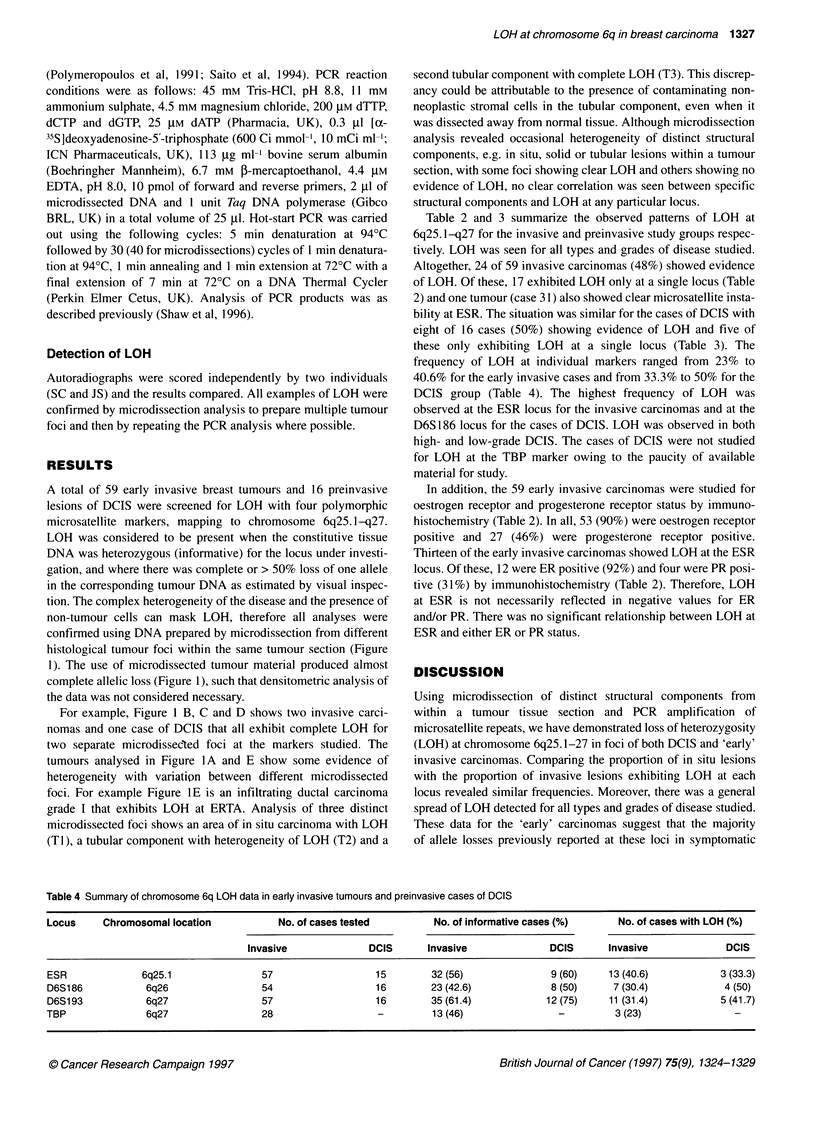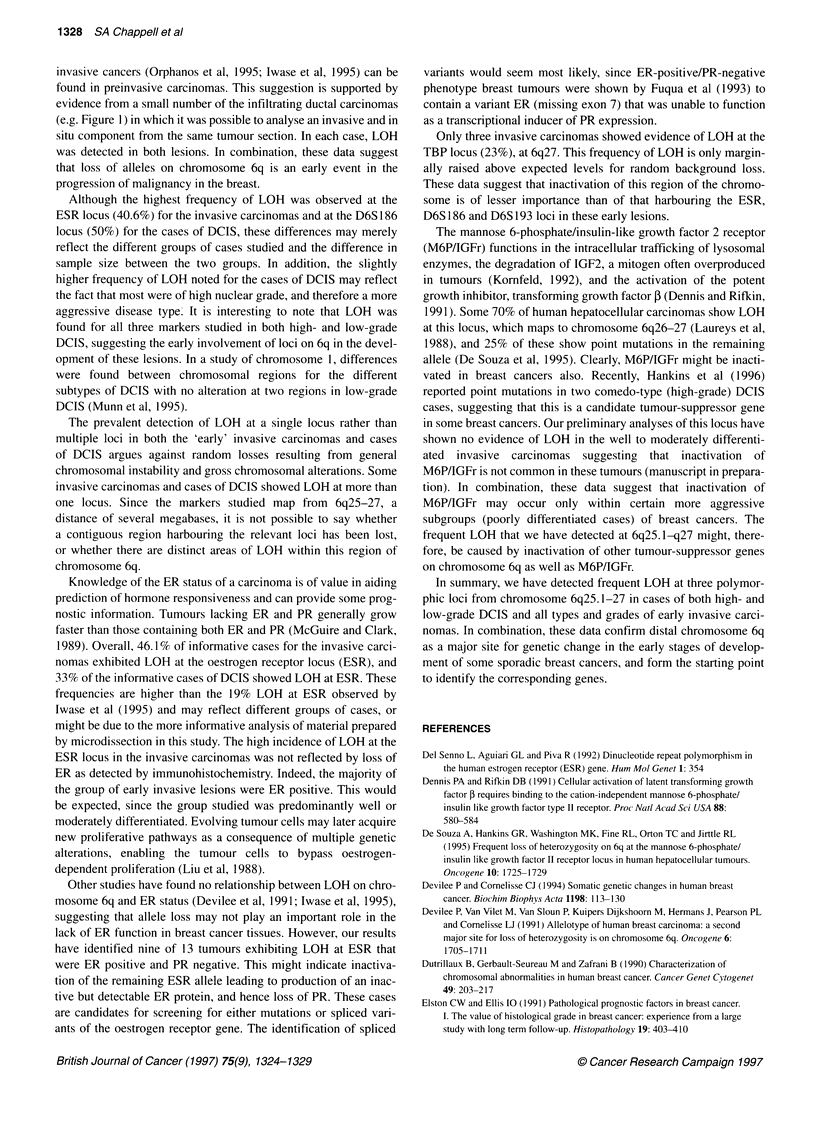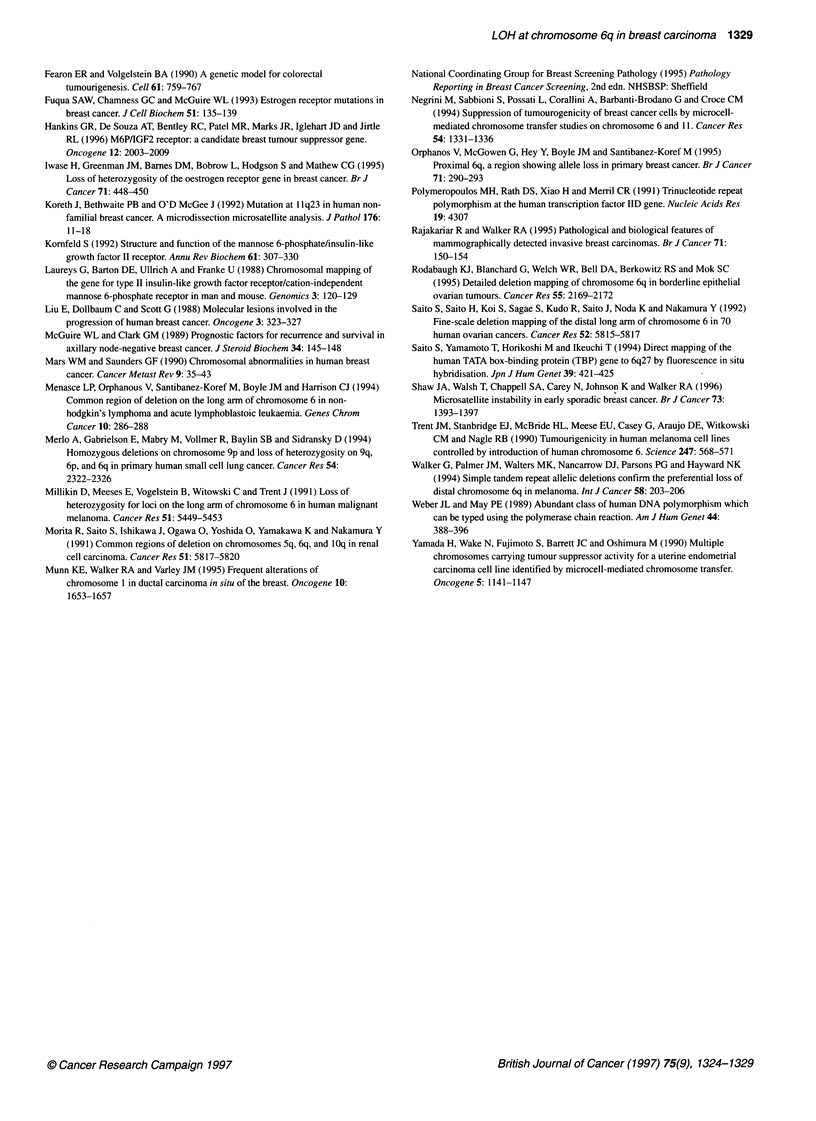# Loss of heterozygosity at chromosome 6q in preinvasive and early invasive breast carcinomas.

**DOI:** 10.1038/bjc.1997.224

**Published:** 1997

**Authors:** S. A. Chappell, T. Walsh, R. A. Walker, J. A. Shaw

**Affiliations:** Department of Pathology, University of Leicester, Glenfield General Hospital, UK.

## Abstract

**Images:**


British Journal of Cancer (1997) 75(9), 1324–1329
© 1997 Cancer Research Campaign

### Loss of heterozygosity at chromosome 6q in preinvasive and early invasive breast carcinomas

**SA Chappell, T Walsh, RA Walker and JA Shaw**

Breast Cancer Research Unit, Department of Pathology, University of Leicester, Clinical Sciences Building, Glenfield General Hospital, Groby Road, Leicester LE3 9QP, UK

**Summary** We have used polymerase chain reaction (PCR) analysis to study the incidence of allelic imbalance at four polymorphic microsatellite markers on chromosome 6q25.1–27, three dinucleotide repeats and one trinucleotide repeat, for microdissected tumour foci from a group of 75 'early' breast carcinomas. The tumours comprised 16 preinvasive cases of ductal carcinoma in situ (DCIS) and 59 mammographically detected early invasive carcinomas. Loss of heterozygosity (LOH) was detected at all four loci and in all types and grade of disease. The frequency of LOH ranged from 23% to 50% depending on the marker studied. The highest frequency of LOH was observed at the D6S186 locus for the cases of DCIS and at the oestrogen receptor locus for the invasive carcinomas. These data suggest that the inactivation of tumour-suppressor genes within this region on chromosome 6q is important for the development of these early lesions.

**Keywords:** breast carcinoma; mammography; loss of heterozygosity; tumour-suppressor genes

According to the multistep model of carcinogenesis, tumours may develop and progress as a result of alterations in oncogene and tumour-suppressor gene loci. In colon cancer, a benign to malignant progression with recognizable molecular changes has been described (Fearon and Volgelstein, 1990). However, the situation for breast cancer is less clear, since there is no clear understanding of the natural history of the disease.

Cytogenetic analyses of primary breast tumours have identified frequent alterations to a number of chromosomes, notably deletions, suggesting the potential localization of tumour-suppressor genes (for a review, see Devilee and Cornelisse, 1994). These studies demonstrated that deletion of chromosome 6q was one of the most frequent chromosomal changes (Dutrillaux et al, 1990; Mars and Saunders, 1990). A subsequent study, using Southern analysis of restriction fragment length polymorphisms to compare constitutional and tumour DNAs, identified chromosome 6q as the second most frequent site for allelic loss (loss of heterozygosity) after 17p in breast cancer (Deville et al, 1991).

Other evidence for the presence of putative tumour-suppressor genes on chromosome 6q comes from chromosome-mediated transfer experiments of normal chromosome 6 into melanoma cell lines (Trent et al, 1990), uterine endometrial cell lines (Yamada et al, 1990) and the breast cancer cell lines, MDA-MD231 and MCF-7 (Negrini et al, 1994), all resulting in the suppression of tumorigenesis.

The advent of polymerase chain reaction (PCR) analysis of microsatellite polymorphisms (Weber and May, 1989) has confirmed the cytogenetic evidence for chromosomal deletion at 6q and has enabled construction of a more detailed deletion map. Allelic loss at 6q24–27 has been observed in different tumour types, including breast carcinoma (Orphanos et al, 1995), ovarian carcinoma (Saito et al, 1992; Rodabaugh et al, 1995), hepatic carcinoma (De Souza et al, 1995), small-cell lung carcinoma (Merlo et al, 1994), renal cell carcinoma (Morita et al, 1991), malignant melanoma (Millikin et al, 1991; Walker et al, 1994) and non-Hodgkin's lymphoma (Menasce et al, 1994). The reported frequencies of allelic loss range from 30% to 60% depending on the tumour types and markers studied. This shared region of allelic loss may harbour putative tumour-suppressor genes that are pleiotropic for these tumour types and reflect a common mechanism of tumorigenesis.

Recent detailed analyses of microsatellite markers on chromosome 6q in breast cancers have highlighted two key regions showing high levels of LOH at 6q13 and 6q26–27, indicating the presence of at least two tumour-suppressor genes (Devilee et al, 1991; Orphanos et al, 1995). Since these studies were concerned with symptomatic, well-established breast carcinomas, it is not clear whether allelic loss on chromosome 6q is an early event in the development of breast cancers. Small, mammographically detected breast cancers form a useful group for study of the involvement of tumour-suppressor genes in tumour development and earlier stages of progression. In this report, we examined LOH at the more distal region, chromosome 6q25–q27, using four polymorphic microsatellite markers, in a group of 75 'early' lesions comprising 59 mammographically detected invasive carcinomas and 16 preinvasive lesions of ductal carcinoma in situ (DCIS).

The markers span a chromosomal region of approximately 15 Mb. Two of the markers (D6S186 and D6S193) were analysed previously in well-established carcinomas (Orphanos et al, 1995). The two other markers studied comprise repeats at or within coding sequences that might be candidates for 'early' mutations in breast cancer: a $(TA)_n$ repeat positioned 1 kb upstream of the oestrogen receptor gene (ESR) (Del Senno et al, 1992) and a $(CAG)_n$ repeat within the human TATA box-binding protein (TBP) (Polymeropoulos et al, 1991). We have analysed the frequency of LOH in the two groups of cases and correlated these data with oestrogen receptor (ER) and progesterone receptor (PR) status and other clinicopathological findings.

*Received 31 July 1996*
*Revised 31 October 1996*
*Accepted 18 November 1996*

*Correspondence to:* JA Shaw

**Table 1** Clinicopathological features of 59 mammographically detected early invasive breast carcinomas

| Type | Grade | Number of cases | Tumour size (mm) | Number of cases |
|---|---|---|---|---|
| Tub | I | 6 | < 10 | 10 |
| Lob/tub | I | 1 | 10 | 14 |
| IDC/ILC | II | 1 | 11 | 4 |
| ILC | II | 1 | 12 | 5 |
| IDC | I | 17 (1) | 13 | 3 |
| IDC | II | 29 (2) | 14 | 2 |
| IDC | III | 4 | 15 | 21 |
| Total | | 59 | | 59 |

Tub, tubular carcinoma; Lob/tub, lobular and tubular carcinoma; IDC/ILC, infiltrating ductal with infiltrating lobular carcinoma; ILC, infiltrating lobular carcinoma; IDC, infiltrating ductal carcinoma. Numbers in brackets, node-positive cases.

### MATERIALS AND METHODS

### Patients

A total of 59 invasive breast carcinomas that were impalpable and detected by mammography were studied. All were from the prevalent round of screening and were detected by the Leicestershire Breast Screening Service. Cases of 15 mm or less in maximum diameter were examined. All had either axillary node sampling or axillary dissection. None of the tumours were from women with either a strong family history of breast cancer or any known inherited predisposition to the development of tumours. Some 56 cases were node negative.

A total of 16 cases of pure ductal carcinoma in situ (DCIS) were studied. These comprised three low, three intermediate and ten high nuclear grade cases. Ten of these were mammographically detected and six were clinically presenting.

### Tissues

All tissues were fixed in 4% formaldehyde in saline for 18–36 h. After slicing, selected blocks were processed through graded alcohols and xylene to paraffin wax. Following review of haematoxylin and eosin-stained sections, representative blocks were chosen for further study. Tissue from histologically normal lymph node served as the source of normal DNA.

### Histology

All carcinomas were reported according to the NHS Breast Screening Programme National Coordinating Group for Breast Screening Pathology Guidelines (1995). Infiltrating ductal carcinomas were graded using the modified Bloom and Richardson system (Elston and Ellis, 1991). Cases of DCIS were graded as low, intermediate or high nuclear grade. All histology was undertaken by RA Walker. The clinicopathological features of the invasive carcinomas are shown in Table 1.

### Oestrogen receptor and progesterone receptor immunohistochemistry

Avidin–biotin complex peroxidase immunohistochemistry was carried out for the 59 early invasive carcinomas as described

**Figure 1** Loss of heterozygosity in early breast cancer. Genomic DNA samples from paired normal lymph node (N) and microdissected tumour (T) samples were compared by PCR amplification, electrophoresis on 6% sequencing gels and autoradiography. (**A**) LOH at D6S193 in case 37: (T1–T3) solid component of infiltrating ductal carcinoma. (**B**) LOH at D6S186 in case 21: (T1 and T2) solid component of infiltrating ductal carcinoma. (**C**) LOH at D6S193 in case 102: (T1 and T2) solid component of infiltrating ductal carcinoma. (**D**) LOH at D6S186 in DCIS case D5: (T1 and T2) individual ducts. (**E**) LOH at ERTA in case 57: (T1) area of DCIS, (T2) tubular component, (T3) tubular component

© Cancer Research Campaign 1997

**Table 2** Pattern of loss of heterozygosity observed using four microsatellite markers from 59 early invasive breast carcinomas

| Case no. | Type | Grade | Loss of heterozygosity at markers | | | | H scores | |
|---|---|---|---|---|---|---|---|---|
| | | | ESR (q25.1) | D6S186 (q26) | D6S193 (q27) | TBP (q27) | ER | PR |
| 3 | ILC | II | ● | ○ | NI | ○ | 175 | 106 |
| 7 | IDC | I | ● | NI | ○ | ○ | 182 | 49 |
| 13 | Tub | I | ○ | ○ | NI | ● | 142 | 94 |
| 15 | IDC | I | ● | NI | NI | ○ | 181 | 97 |
| 17 | Tub | I | ○ | ○ | NI | ● | 195 | 3 |
| 19 | IDC | II | ○ | NI | ● | ○ | 162 | 168 |
| 21 | IDC | II | ● | ● | ● | NI | 151 | 92 |
| 23 | IDC | I | ● | ● | NI | NA | 175 | 31 |
| 29 | Lob/tub | I | ● | NI | ○ | NI | 196 | 0 |
| 31 | IDC | I | MSI | NI | NI | ● | 202 | 115 |
| 37 | IDC | II | NI | ○ | ● | ○ | 22 | 3 |
| 41 | IDC | II | NI | ○ | ● | ○ | 192 | 170 |
| 49 | IDC | II | NI | ● | NI | NI | 215 | 46 |
| 57 | IDC | I | ● | NI | ○ | NT | 173 | 192 |
| 59 | IDC | I | ● | ● | ● | NI | 196 | 19.2 |
| 70 | IDC | I | ● | NI | NA | NI | 231 | 0 |
| 76 | IDC | I | ● | NI | NA | NI | 219 | 0 |
| 78 | IDC | I | NI | ● | ○ | NT | 202.5 | 85 |
| 80 | Tub | I | NI | NI | ● | NI | 162 | 83 |
| 98 | IDC | II | ● | ● | ● | NT | 187.5 | 0 |
| 102 | IDC | II | ● | ● | ● | NT | 142 | 0 |
| 106 | IDC | II | ○ | NI | ● | NT | 195 | 33.5 |
| 108 | IDC | II | ● | NI | ● | NI | 0 | 0 |
| 122 | IDC | II | NI | NI | ● | NI | 160 | 0 |

●, loss of heterozygosity; ○, heterozygosity; MSI, microsatellite instability; NI, not informative; NA, no amplification; NT, not tested, Tub, tubular carcinoma; Lob/tub, lobular and tubular carcinoma; ILC, infiltrating lobular carcinoma; IDC, infiltrating ductal carcinoma.

**Table 3** Pattern of loss of heterozygosity observed using three microsatellite markers from 16 preinvasive lesions of DCIS

| Case no. | Grade | Chromosome 6q markers | | | |
|---|---|---|---|---|---|
| | | ESR (q25.1) | D6S186 (q26) | D6S193 (q27) | TBP (q27) |
| D2 (M) | High | NA | ● | NI | NT |
| D3 (M) | Low | ● | NI | ○ | NT |
| D4 (M) | Low | NI | ● | ● | NT |
| D5 (M) | High | ● | ● | ● | NT |
| D8 (M) | Low | NI | NI | ● | NT |
| D12 (C) | High | NI | ● | ● | NT |
| D13 (C) | High | ● | NI | ○ | NT |
| D14 (C) | High | NI | NI | ● | NT |

●, loss of heterozygosity; ○, heterozygosity; NI, not informative; NA, no amplification; NT, not tested; (M), mammographically detected; (C), clinically presenting.

previously (Rajakariar and Walker, 1995) with minor modifications. For antigen retrieval pretreatment, sections were exposed to two cycles of microwaving for oestrogen receptor [mouse monoclonal ID5 (Dako)] and progesterone receptor [mouse monoclonal NCL-PgR (NovaCastra)].

### DNA extraction and microdissection from paraffin embedded sections

Formalin-fixed, paraffin-embedded tissue from breast tumour samples and non-involved lymph nodes served as the source of tumour and normal DNA respectively. For each tumour–normal pair, DNA was extracted from 10-μm paraffin-embedded sections as described previously (Shaw et al, 1996).

Microdissection of tumour foci from invasive carcinomas and areas of DCIS was carried out using a method based on that described by Koreth et al (1995). In brief, serial 10-μm paraffin sections were deparaffinized in xylene (2 × 5 min) and dehydrated in 99% ethanol (2 × 2 min) and 95% ethanol (1 × 2 min), and rehydrated in water before staining. Tissues were stained in 0.5% eosin solution for 20 s, washed in water and allowed to air dry. A serial reference slide for each tumour was stained with haematoxylin and eosin, dehydrated and coverslipped. Tumour foci of interest from the invasive carcinomas included tubular, solid, invasive lobular components and preinvasive areas of ductal carcinoma in situ (DCIS) within infiltrating ductal carcinomas. These areas were visualized using the haematoxylin and eosin reference and microdissected from corresponding eosin sections using a × 40 magnification microdissection microscope (American Optical Corporation) using sterile, 20-μl drawn-out glass Pasteur pipettes. Tumour foci (approximately 100 cells) were placed into 25 μl of digestion buffer [100 mM Tris-HCl (pH 7.6), 1 mM EDTA (pH 8), 200 μg $ml^{-1}$ proteinase K] and incubated at 55°C for 3 h, then at 94°C for 10 min. Volumes (2 μl) of this mixture were used in the PCR analysis.

### PCR analysis at 6q25.1–27

A total of 75 'early' breast carcinomas were studied for LOH at four polymorphic markers from chromosome 6q25.1–27: the oestrogen receptor (ESR) at 6q25.1 (Del Senno et al, 1992), D6S186 (6q26) and D6S193 (6q27) (Saito et al, 1992; Orphanos et al, 1995) and the TATA box-binding protein (TBP) gene at 6q27

*© Cancer Research Campaign 1997*

(Polymeropoulos et al, 1991; Saito et al, 1994). PCR reaction conditions were as follows: 45 mM Tris-HCl, pH 8.8, 11 mM ammonium sulphate, 4.5 mM magnesium chloride, 200 μM dTTP, dCTP and dGTP, 25 μM dATP (Pharmacia, UK), 0.3 μl [α-$^{35}$S]deoxyadenosine-5′-triphosphate (600 Ci mmol$^{-1}$, 10 mCi ml$^{-1}$; ICN Pharmaceuticals, UK), 113 μg ml$^{-1}$ bovine serum albumin (Boehringher Mannheim), 6.7 mM β-mercaptoethanol, 4.4 μM EDTA, pH 8.0, 10 pmol of forward and reverse primers, 2 μl of microdissected DNA and 1 unit *Taq* DNA polymerase (Gibco BRL, UK) in a total volume of 25 μl. Hot-start PCR was carried out using the following cycles: 5 min denaturation at 94°C followed by 30 (40 for microdissections) cycles of 1 min denaturation at 94°C, 1 min annealing and 1 min extension at 72°C with a final extension of 7 min at 72°C on a DNA Thermal Cycler (Perkin Elmer Cetus, UK). Analysis of PCR products was as described previously (Shaw et al, 1996).

### Detection of LOH

Autoradiographs were scored independently by two individuals (SC and JS) and the results compared. All examples of LOH were confirmed by microdissection analysis to prepare multiple tumour foci and then by repeating the PCR analysis where possible.

### RESULTS

A total of 59 early invasive breast tumours and 16 preinvasive lesions of DCIS were screened for LOH with four polymorphic microsatellite markers, mapping to chromosome 6q25.1–q27. LOH was considered to be present when the constitutive tissue DNA was heterozygous (informative) for the locus under investigation, and where there was complete or > 50% loss of one allele in the corresponding tumour DNA as estimated by visual inspection. The complex heterogeneity of the disease and the presence of non-tumour cells can mask LOH, therefore all analyses were confirmed using DNA prepared by microdissection from different histological tumour foci within the same tumour section (Figure 1). The use of microdissected tumour material produced almost complete allelic loss (Figure 1), such that densitometric analysis of the data was not considered necessary.

For example, Figure 1 B, C and D shows two invasive carcinomas and one case of DCIS that all exhibit complete LOH for two separate microdissected foci at the markers studied. The tumours analysed in Figure 1A and E show some evidence of heterogeneity with variation between different microdissected foci. For example Figure 1E is an infiltrating ductal carcinoma grade I that exhibits LOH at ERTA. Analysis of three distinct microdissected foci shows an area of in situ carcinoma with LOH (T1), a tubular component with heterogeneity of LOH (T2) and a second tubular component with complete LOH (T3). This discrepancy could be attributable to the presence of contaminating non-neoplastic stromal cells in the tubular component, even when it was dissected away from normal tissue. Although microdissection analysis revealed occasional heterogeneity of distinct structural components, e.g. in situ, solid or tubular lesions within a tumour section, with some foci showing clear LOH and others showing no evidence of LOH, no clear correlation was seen between specific structural components and LOH at any particular locus.

Table 2 and 3 summarize the observed patterns of LOH at 6q25.1–q27 for the invasive and preinvasive study groups respectively. LOH was seen for all types and grades of disease studied. Altogether, 24 of 59 invasive carcinomas (48%) showed evidence of LOH. Of these, 17 exhibited LOH only at a single locus (Table 2) and one tumour (case 31) also showed clear microsatellite instability at ESR. The situation was similar for the cases of DCIS with eight of 16 cases (50%) showing evidence of LOH and five of these only exhibiting LOH at a single locus (Table 3). The frequency of LOH at individual markers ranged from 23% to 40.6% for the early invasive cases and from 33.3% to 50% for the DCIS group (Table 4). The highest frequency of LOH was observed at the ESR locus for the invasive carcinomas and at the D6S186 locus for the cases of DCIS. LOH was observed in both high- and low-grade DCIS. The cases of DCIS were not studied for LOH at the TBP marker owing to the paucity of available material for study.

In addition, the 59 early invasive carcinomas were studied for oestrogen receptor and progesterone receptor status by immunohistochemistry (Table 2). In all, 53 (90%) were oestrogen receptor positive and 27 (46%) were progesterone receptor positive. Thirteen of the early invasive carcinomas showed LOH at the ESR locus. Of these, 12 were ER positive (92%) and four were PR positive (31%) by immunohistochemistry (Table 2). Therefore, LOH at ESR is not necessarily reflected in negative values for ER and/or PR. There was no significant relationship between LOH at ESR and either ER or PR status.

### DISCUSSION

Using microdissection of distinct structural components from within a tumour tissue section and PCR amplification of microsatellite repeats, we have demonstrated loss of heterozygosity (LOH) at chromosome 6q25.1–27 in foci of both DCIS and 'early' invasive carcinomas. Comparing the proportion of in situ lesions with the proportion of invasive lesions exhibiting LOH at each locus revealed similar frequencies. Moreover, there was a general spread of LOH detected for all types and grades of disease studied. These data for the 'early' carcinomas suggest that the majority of allele losses previously reported at these loci in symptomatic

**Table 4** Summary of chromosome 6q LOH data in early invasive tumours and preinvasive cases of DCIS

| Locus | Chromosomal location | No. of cases tested | | No. of informative cases (%) | | No. of cases with LOH (%) | |
|---|---|---|---|---|---|---|---|
| | | Invasive | DCIS | Invasive | DCIS | Invasive | DCIS |
| ESR | 6q25.1 | 57 | 15 | 32 (56) | 9 (60) | 13 (40.6) | 3 (33.3) |
| D6S186 | 6q26 | 54 | 16 | 23 (42.6) | 8 (50) | 7 (30.4) | 4 (50) |
| D6S193 | 6q27 | 57 | 16 | 35 (61.4) | 12 (75) | 11 (31.4) | 5 (41.7) |
| TBP | 6q27 | 28 | – | 13 (46) | – | 3 (23) | – |

© Cancer Research Campaign 1997

invasive cancers (Orphanos et al, 1995; Iwase et al, 1995) can be found in preinvasive carcinomas. This suggestion is supported by evidence from a small number of the infiltrating ductal carcinomas (e.g. Figure 1) in which it was possible to analyse an invasive and in situ component from the same tumour section. In each case, LOH was detected in both lesions. In combination, these data suggest that loss of alleles on chromosome 6q is an early event in the progression of malignancy in the breast.

Although the highest frequency of LOH was observed at the ESR locus (40.6%) for the invasive carcinomas and at the D6S186 locus (50%) for the cases of DCIS, these differences may merely reflect the different groups of cases studied and the difference in sample size between the two groups. In addition, the slightly higher frequency of LOH noted for the cases of DCIS may reflect the fact that most were of high nuclear grade, and therefore a more aggressive disease type. It is interesting to note that LOH was found for all three markers studied in both high- and low-grade DCIS, suggesting the early involvement of loci on 6q in the development of these lesions. In a study of chromosome 1, differences were found between chromosomal regions for the different subtypes of DCIS with no alteration at two regions in low-grade DCIS (Munn et al, 1995).

The prevalent detection of LOH at a single locus rather than multiple loci in both the 'early' invasive carcinomas and cases of DCIS argues against random losses resulting from general chromosomal instability and gross chromosomal alterations. Some invasive carcinomas and cases of DCIS showed LOH at more than one locus. Since the markers studied map from 6q25–27, a distance of several megabases, it is not possible to say whether a contiguous region harbouring the relevant loci has been lost, or whether there are distinct areas of LOH within this region of chromosome 6q.

Knowledge of the ER status of a carcinoma is of value in aiding prediction of hormone responsiveness and can provide some prognostic information. Tumours lacking ER and PR generally grow faster than those containing both ER and PR (McGuire and Clark, 1989). Overall, 46.1% of informative cases for the invasive carcinomas exhibited LOH at the oestrogen receptor locus (ESR), and 33% of the informative cases of DCIS showed LOH at ESR. These frequencies are higher than the 19% LOH at ESR observed by Iwase et al (1995) and may reflect different groups of cases, or might be due to the more informative analysis of material prepared by microdissection in this study. The high incidence of LOH at the ESR locus in the invasive carcinomas was not reflected by loss of ER as detected by immunohistochemistry. Indeed, the majority of the group of early invasive lesions were ER positive. This would be expected, since the group studied was predominantly well or moderately differentiated. Evolving tumour cells may later acquire new proliferative pathways as a consequence of multiple genetic alterations, enabling the tumour cells to bypass oestrogen-dependent proliferation (Liu et al, 1988).

Other studies have found no relationship between LOH on chromosome 6q and ER status (Devilee et al, 1991; Iwase et al, 1995), suggesting that allele loss may not play an important role in the lack of ER function in breast cancer tissues. However, our results have identified nine of 13 tumours exhibiting LOH at ESR that were ER positive and PR negative. This might indicate inactivation of the remaining ESR allele leading to production of an inactive but detectable ER protein, and hence loss of PR. These cases are candidates for screening for either mutations or spliced variants of the oestrogen receptor gene. The identification of spliced variants would seem most likely, since ER-positive/PR-negative phenotype breast tumours were shown by Fuqua et al (1993) to contain a variant ER (missing exon 7) that was unable to function as a transcriptional inducer of PR expression.

Only three invasive carcinomas showed evidence of LOH at the TBP locus (23%), at 6q27. This frequency of LOH is only marginally raised above expected levels for random background loss. These data suggest that inactivation of this region of the chromosome is of lesser importance than of that harbouring the ESR, D6S186 and D6S193 loci in these early lesions.

The mannose 6-phosphate/insulin-like growth factor 2 receptor (M6P/IGFr) functions in the intracellular trafficking of lysosomal enzymes, the degradation of IGF2, a mitogen often overproduced in tumours (Kornfeld, 1992), and the activation of the potent growth inhibitor, transforming growth factor β (Dennis and Rifkin, 1991). Some 70% of human hepatocellular carcinomas show LOH at this locus, which maps to chromosome 6q26–27 (Laureys et al, 1988), and 25% of these show point mutations in the remaining allele (De Souza et al, 1995). Clearly, M6P/IGFr might be inactivated in breast cancers also. Recently, Hankins et al (1996) reported point mutations in two comedo-type (high-grade) DCIS cases, suggesting that this is a candidate tumour-suppressor gene in some breast cancers. Our preliminary analyses of this locus have shown no evidence of LOH in the well to moderately differentiated invasive carcinomas suggesting that inactivation of M6P/IGFr is not common in these tumours (manuscript in preparation). In combination, these data suggest that inactivation of M6P/IGFr may occur only within certain more aggressive subgroups (poorly differentiated cases) of breast cancers. The frequent LOH that we have detected at 6q25.1–q27 might, therefore, be caused by inactivation of other tumour-suppressor genes on chromosome 6q as well as M6P/IGFr.

In summary, we have detected frequent LOH at three polymorphic loci from chromosome 6q25.1–27 in cases of both high- and low-grade DCIS and all types and grades of early invasive carcinomas. In combination, these data confirm distal chromosome 6q as a major site for genetic change in the early stages of development of some sporadic breast cancers, and form the starting point to identify the corresponding genes.

### REFERENCES

Del Senno L, Aguiari GL and Piva R (1992) Dinucleotide repeat polymorphism in the human estrogen receptor (ESR) gene. *Hum Mol Genet* **1**: 354

Dennis PA and Rifkin DB (1991) Cellular activation of latent transforming growth factor β requires binding to the cation-independent mannose 6-phosphate/insulin like growth factor type II receptor. *Proc Natl Acad Sci USA* **88**: 580–584

De Souza A, Hankins GR, Washington MK, Fine RL, Orton TC and Jirtle RL (1995) Frequent loss of heterozygosity on 6q at the mannose 6-phosphate/insulin like growth factor II receptor locus in human hepatocellular tumours. *Oncogene* **10**: 1725–1729

Devilee P and Cornelisse CJ (1994) Somatic genetic changes in human breast cancer. *Biochim Biophys Acta* **1198**: 113–130

Devilee P, Van Vilet M, Van Sloun P, Kuipers Dijkshoorn M, Hermans J, Pearson PL and Cornelisse LJ (1991) Allelotype of human breast carcinoma: a second major site for loss of heterozygosity is on chromosome 6q. *Oncogene* **6**: 1705–1711

Dutrillaux B, Gerbault-Seureau M and Zafrani B (1990) Characterization of chromosomal abnormalities in human breast cancer. *Cancer Genet Cytogenet* **49**: 203–217

Elston CW and Ellis IO (1991) Pathological prognostic factors in breast cancer. I. The value of histological grade in breast cancer: experience from a large study with long term follow-up. *Histopathology* **19**: 403–410

© Cancer Research Campaign 1997

Fearon ER and Volgelstein BA (1990) A genetic model for colorectal tumourigenesis. *Cell* **61**: 759–767

Fuqua SAW, Chamness GC and McGuire WL (1993) Estrogen receptor mutations in breast cancer. *J Cell Biochem* **51**: 135–139

Hankins GR, De Souza AT, Bentley RC, Patel MR, Marks JR, Iglehart JD and Jirtle RL (1996) M6P/IGF2 receptor: a candidate breast tumour suppressor gene. *Oncogene* **12**: 2003–2009

Iwase H, Greenman JM, Barnes DM, Bobrow L, Hodgson S and Mathew CG (1995) Loss of heterozygosity of the oestrogen receptor gene in breast cancer. *Br J Cancer* **71**: 448–450

Koreth J, Bethwaite PB and O'D McGee J (1992) Mutation at 11q23 in human non-familial breast cancer. A microdissection microsatellite analysis. *J Pathol* **176**: 11–18

Kornfeld S (1992) Structure and function of the mannose 6-phosphate/insulin-like growth factor II receptor. *Annu Rev Biochem* **61**: 307–330

Laureys G, Barton DE, Ullrich A and Franke U (1988) Chromosomal mapping of the gene for type II insulin-like growth factor receptor/cation-independent mannose 6-phosphate receptor in man and mouse. *Genomics* **3**: 120–129

Liu E, Dollbaum C and Scott G (1988) Molecular lesions involved in the progression of human breast cancer. *Oncogene* **3**: 323–327

McGuire WL and Clark GM (1989) Prognostic factors for recurrence and survival in axillary node-negative breast cancer. *J Steroid Biochem* **34**: 145–148

Mars WM and Saunders GF (1990) Chromosomal abnormalities in human breast cancer. *Cancer Metast Rev* **9**: 35–43

Menasce LP, Orphanous V, Santibanez-Koref M, Boyle JM and Harrison CJ (1994) Common region of deletion on the long arm of chromosome 6 in non-hodgkin's lymphoma and acute lymphoblastoic leukaemia. *Genes Chrom Cancer* **10**: 286–288

Merlo A, Gabrielson E, Mabry M, Vollmer R, Baylin SB and Sidransky D (1994) Homozygous deletions on chromosome 9p and loss of heterozygosity on 9q, 6p, and 6q in primary human small cell lung cancer. *Cancer Res* **54**: 2322–2326

Millikin D, Meeses E, Vogelstein B, Witowski C and Trent J (1991) Loss of heterozygosity for loci on the long arm of chromosome 6 in human malignant melanoma. *Cancer Res* **51**: 5449–5453

Morita R, Saito S, Ishikawa J, Ogawa O, Yoshida O, Yamakawa K and Nakamura Y (1991) Common regions of deletion on chromosomes 5q, 6q, and 10q in renal cell carcinoma. *Cancer Res* **51**: 5817–5820

Munn KE, Walker RA and Varley JM (1995) Frequent alterations of chromosome 1 in ductal carcinoma *in situ* of the breast. *Oncogene* **10**: 1653–1657

National Coordinating Group for Breast Screening Pathology (1995) *Pathology Reporting in Breast Cancer Screening*, 2nd edn. NHSBSP: Sheffield

Negrini M, Sabbioni S, Possati L, Corallini A, Barbanti-Brodano G and Croce CM (1994) Suppression of tumourogenicity of breast cancer cells by microcell-mediated chromosome transfer studies on chromosome 6 and 11. *Cancer Res* **54**: 1331–1336

Orphanos V, McGowen G, Hey Y, Boyle JM and Santibanez-Koref M (1995) Proximal 6q, a region showing allele loss in primary breast cancer. *Br J Cancer* **71**: 290–293

Polymeropoulos MH, Rath DS, Xiao H and Merril CR (1991) Trinucleotide repeat polymorphism at the human transcription factor IID gene. *Nucleic Acids Res* **19**: 4307

Rajakariar R and Walker RA (1995) Pathological and biological features of mammographically detected invasive breast carcinomas. *Br J Cancer* **71**: 150–154

Rodabaugh KJ, Blanchard G, Welch WR, Bell DA, Berkowitz RS and Mok SC (1995) Detailed deletion mapping of chromosome 6q in borderline epithelial ovarian tumours. *Cancer Res* **55**: 2169–2172

Saito S, Saito H, Koi S, Sagae S, Kudo R, Saito J, Noda K and Nakamura Y (1992) Fine-scale deletion mapping of the distal long arm of chromosome 6 in 70 human ovarian cancers. *Cancer Res* **52**: 5815–5817

Saito S, Yamamoto T, Horikoshi M and Ikeuchi T (1994) Direct mapping of the human TATA box-binding protein (TBP) gene to 6q27 by fluorescence in situ hybridisation. *Jpn J Hum Genet* **39**: 421–425

Shaw JA, Walsh T, Chappell SA, Carey N, Johnson K and Walker RA (1996) Microsatellite instability in early sporadic breast cancer. *Br J Cancer* **73**: 1393–1397

Trent JM, Stanbridge EJ, McBride HL, Meese EU, Casey G, Araujo DE, Witkowski CM and Nagle RB (1990) Tumourigenicity in human melanoma cell lines controlled by introduction of human chromosome 6. *Science* **247**: 568–571

Walker G, Palmer JM, Walters MK, Nancarrow DJ, Parsons PG and Hayward NK (1994) Simple tandem repeat allelic deletions confirm the preferential loss of distal chromosome 6q in melanoma. *Int J Cancer* **58**: 203–206

Weber JL and May PE (1989) Abundant class of human DNA polymorphism which can be typed using the polymerase chain reaction. *Am J Hum Genet* **44**: 388–396

Yamada H, Wake N, Fujimoto S, Barrett JC and Oshimura M (1990) Multiple chromosomes carrying tumour suppressor activity for a uterine endometrial carcinoma cell line identified by microcell-mediated chromosome transfer. *Oncogene* **5**: 1141–1147

© Cancer Research Campaign 1997